# Effectiveness of pharmacist home visits for individuals at risk of medication-related problems: a systematic review and meta-analysis of randomised controlled trials

**DOI:** 10.1186/s12913-019-4728-3

**Published:** 2020-01-15

**Authors:** Rebecca A. Abbott, Darren A. Moore, Morwenna Rogers, Alison Bethel, Ken Stein, Jo Thompson Coon

**Affiliations:** 1 Evidence Synthesis Team, PenCLAHRC University of Exeter Medical School, St Luke’s Campus, Exeter, EX1 2LU UK; 2 Graduate School of Education, St Luke’s Campus, Exeter, EX1 2LU UK

**Keywords:** Medication review, Pharmacist intervention, Community, Home visit, Older adults, Hospital admission, Randomised controlled trials, Systematic review

## Abstract

**Background:**

Medication mismanagement is a major cause of both hospital admission and nursing home placement of frail older adults. Medication reviews by community pharmacists aim to maximise therapeutic benefit but also minimise harm. Pharmacist-led medication reviews have been the focus of several systematic reviews, but none have focussed on the home setting.

**Review methods:**

To determine the effectiveness of pharmacist home visits for individuals at risk of medication-related problems we undertook a systematic review and meta-analysis of randomised controlled trials (RCTs). Thirteen databases were searched from inception to December 2018. Forward and backward citation of included studies was also performed. Articles were screened for inclusion independently by two reviewers. Randomised controlled studies of home visits by pharmacists for individuals at risk of medication-related problems were eligible for inclusion. Data extraction and quality appraisal were performed by one reviewer and checked by a second. Random-effects meta-analyses were performed where sufficient data allowed and narrative synthesis summarised all remaining data.

**Results:**

Twelve RCTs (reported in 15 articles), involving 3410 participants, were included in the review. The frequency, content and purpose of the home visit varied considerably. The data from eight trials were suitable for meta-analysis of the effects on hospital admissions and mortality, and from three trials for the effects on quality of life. Overall there was no evidence of reduction in hospital admissions (risk ratio (RR) of 1.01 (95%CI 0.86 to 1.20, I^2^ = 69.0%, *p* = 0.89; 8 studies, 2314 participants)), or mortality (RR of 1.01 (95%CI 0.81 to 1.26, I^2^ = 0%, *p* = 0.94; 8 studies, 2314 participants)). There was no consistent evidence of an effect on quality of life, medication adherence or knowledge.

**Conclusion:**

A systematic review of twelve RCTs assessing the impact of pharmacist home visits for individuals at risk of medication related problems found no evidence of effect on hospital admission or mortality rates, and limited evidence of effect on quality of life. Future studies should focus on using more robust methods to assess relevant outcomes.

## Background

For many older adults, the ability to remain independent in one’s home depends on the ability to manage medication. Medication mismanagement and drug-related problems are a major cause of nursing home placement of frail older adults [1]. Studies from across Europe have estimated that the proportion of elderly experiencing drug related problems that lead to hospital admission range between 4 and 30% [2]. Furthermore, research indicates that for older adults, more than half of hospital admissions for adverse drug reactions are preventable, with less than a third considered unavoidable [3].

In the United Kingdom (UK), the National Service for Older People Framework [4] recommends regular medication reviews for people > 75 yrs. This is not only to maximise therapeutic benefit but also to minimise harm. Medication reviews can vary from brief opportunistic reviews of drug doses to full clinical medication reviews which may involve: medication education, assessing the clinical appropriateness of the drug regimen, the potential and evidence for drug interactions, the patients’ understanding of the drugs and compliance to recommended doses, the ability to take medicines as prescribed, assessing the storage of medicines and removal of unnecessary or out of date medicines [5]. A ‘Medicines Use Review’, a free National Health Service offered by pharmacies in the UK, was introduced in 2005 [6]. Whilst this is not intended to provide a full medication review service, it is intended to improve the patient’s knowledge and use of drugs, as well as identify drug-related problems (DRPs). In the UK, and internationally, medication reviews are increasingly being commissioned through community pharmacies as they are seen to support patient medicine adherence [7]. In Australia, the Home Medicines Review programme stipulates a home visit as part of the service [8]. However, in the UK home medicine reviews are typically held within the pharmacy and although home visits can be made for exceptional cases, they are currently not routine.

Many elderly patients however are unable to attend their pharmacy or primary care centre for advice. A UK study of 1000 patients over 75 years of age, and taking four or more prescriptions, reported that 58% could not collect their prescriptions in person due to issues such as being housebound, having poor eyesight or being unable to walk the distance [9]. Estimates of the prevalence of housebound people vary from 4.7 to 19.5% [10, 11]. Whilst a recent systematic review of preventative home visits (primary, secondary and tertiary prevention interventions) for community-dwelling older adults [12] found no effect on independent living, hospital admission or mortality, the studies varied considerably in focus and goals, healthcare professional involved and type of intervention, with the predominant interventions being falls related, and nurse-led. Pharmacist-led interventions were not identified or considered separately. Pharmacist-led medication reviews have been the focus of several systematic reviews, but none have focussed on the home setting [13–15]. It has been suggested that medication reviews undertaken in the home may facilitate identification of medicine issues and may provide a more receptive environment in which to provide medication advice and education [16].

The aim of this systematic review therefore was to assess whether pharmacist home visits, for the purpose of medication review are effective in improving the health of individuals at risk of medication-related problems.

## Methods

The systematic review was conducted following the general principles published by the Centre for Reviews and Dissemination [17] and is reported according to the Preferred Reporting Items for Systematic Reviews and Meta Analyses (PRISMA) statement [18]. The protocol for this review was developed in consultation with two experts in community pharmacy and registered with the International Prospective Register of Systematic Review (CRD42015021965).

### Types of studies

Only RCTs were eligible for inclusion.

### Types of participants

Eligible studies included participants at risk of medication-related problems. Participants had to be living in their own home in the community.

### Types of interventions

Interventions that were described as a home visit service by pharmacists were eligible for inclusion. The purpose of the visit had to be to identify medication-related problems, with or without a full medication review. Multi-professional visits, such as those combining a nurse, pharmacist and general practitioner (GP) were excluded.

### Outcome measures

Outcome measures of interest were: hospital admission/readmission, mortality, medication adherence, changes in medication, quality of life, costs and drug-related adverse events.

### Search strategy

The search strategy was developed by two information specialists (MR, AB) in consultation with topic and methods experts. The strategy used a combination of MeSH terms and free text terms (see Additional file 1). The strategy was developed for MEDLINE and adapted as appropriate for the other searched databases (EMBASE, International Pharmaceutical Abstracts, HMIC Health Management Information Consortium, Social Policy and Practice, and PsycINFO [via OVID]; CDSR and CENTRAL [via The Cochrane Library]; CINAHL, Ageline and AMED [via EBSCOhost]; British Nursing Index [via ProQuest], and the Science Citation Index [via Web of Science]). All databases except International Pharmaceutical Abstracts were searched from inception to October 2017, and updated in December 2018. International Pharmaceutical Abstracts from inception to December 2018. No date or language restrictions were used. Forward and backward citation chasing of each included article was conducted using ISI Web of Knowledge. Two reviewers (RA, MR or AB) independently screened titles and abstracts using the eligibility criteria. Discrepancies were discussed and resolved by a third reviewer (RA, MR or AB) where necessary. Full text screening of remaining abstracts was independently performed by two reviewers (RA, DM). Discrepancies were discussed and resolved by a third reviewer (JTC) where necessary.

### Risk of bias

The methodological quality of each paper was assessed using the Cochrane risk of bias tool [19]. The tool includes six key criteria against which potential risk of bias is judged: adequacy of allocation sequence generation; adequacy of allocation concealment; blinding of participants, personnel or outcome assessors; completeness of outcome data; selectivity of outcome reporting, and other bias. In addition to the Cochrane risk of bias tool, two additional aspects of possible bias were assessed: similarity of baseline characteristics and whether intention to treat analyses were used. Risk of bias was assessed by one reviewer (RA), with judgements checked by a second (MR or DM). Any discrepancies were discussed and resolved.

### Data extraction

Data on the study purpose, population demographics, study inclusion criteria, content and delivery of interventions, primary and secondary outcomes and risk of bias items were extracted from each study. All data were collected using a bespoke data extraction form in Excel, which was first piloted. Data were extracted by one reviewer (RA) and fully checked by another (MR or DM).

### Data analysis

Where sufficient data allowed, meta-analyses were performed. For dichotomous outcomes of hospital admission and death, we calculated the estimated treatment effect as the risk ratio of an event between those in the intervention compared to those in the control arm. We used the last available time point measure in analyses. We calculated pooled risk ratios using the Mantel-Haenszel random effects approach. In addition to incorporating variability within studies, the random effects model also incorporates the variance of treatment effect between studies, which gives the magnitude of heterogeneity of treatment effect. For continuous outcomes, such as quality of life, we calculated the standardised mean difference (SMD). Again, we used a random effects model and the last available time point measured to analyse the differences between intervention and control group means. Effect sizes for the continuous outcomes were calculated using Cohen’s d with Hedge’s correction [20]. I^2^ is estimates the statistical heterogeneity (observed variability in study results that is greater than that expected to occur by chance) and an I^2^ of 70% or more indicates substantial heterogeneity [21].

## Results

**Fig. 1:**
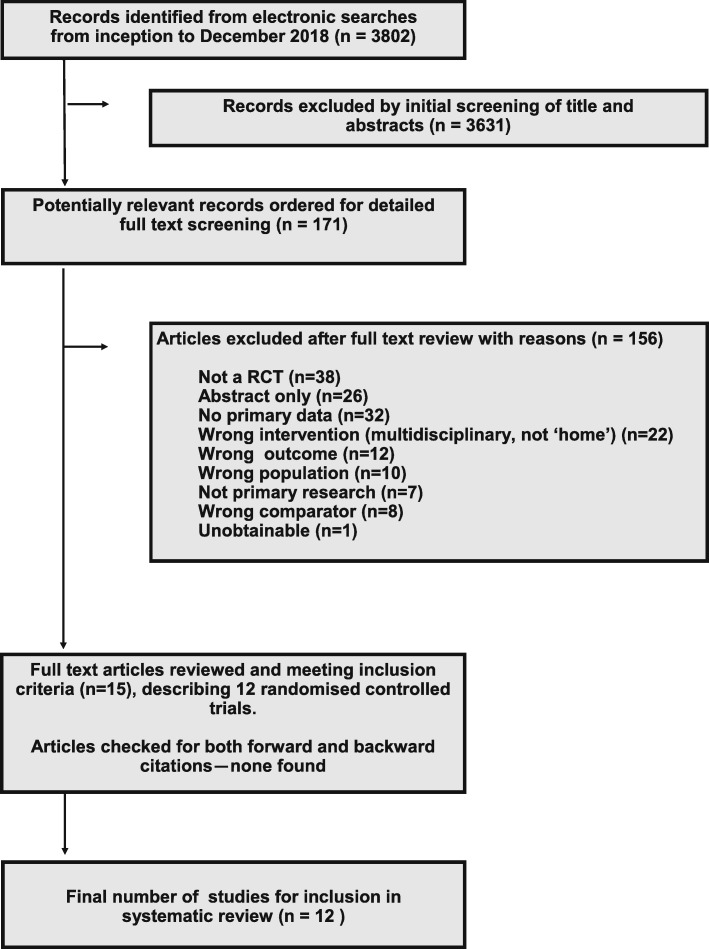
PRISMA flow diagram of article selection

The electronic searches found a total of 3802 articles. After title and abstract screening, 171 full texts were retrieved for closer examination. Of these, 156 were excluded: the reasons for exclusion at the full text stage can be seen in Fig. 1. A total of 12 RCTs (reported in 15 articles) were included in the final review. No additional articles were identified from forward and backward citation chasing. The main trial characteristics are shown in Table 1.

**Table 1:** Main characteristics of pharmacist home visit intervention trials

Study ID	Country	Design (groups) and n (number randomised)	Population, i) Age (yrs) *mean (SD*); ii) Baseline prescribed medications *mean (SD)*	Home visit frequency (INT group only)	Components of home visit	Comparator	Primary Outcome
Begley 1995 [35] (and Begley 1997 [23])	UK	RCT (2 INT, 1 C) *n* = 222	Post-discharge (after emergency admission), Age: INT 84(−); C 82(−) Meds: INT 5 (2); C 5 (2)	Group 1: No counselling Group 2: With counselling 5 visits (2w,1 m,3 m,1y)	Medication knowledge and compliance assessment (Group 1). As above PLUS education on medication management and storage, and advice on improving compliance (Group 2).	Usual care	Medication management
Holland 2005 [24] (Holland 2006 [34], and Pacini 2007 [5])	UK	RCT (1 INT, 1 C) *n* = 822 *The HOMER trial*	Post-discharge (after emergency admission), Age: INT 85.5 (4); C 85.5 (4) Meds: INT 6 (3); 6 (2)	2 visits (within 2 weeks, 6-8 weeks)	Educate, remove out of date drugs, assessed adherence and advised on medication issues. Liaised with GP and local pharmacist where needed	Usual care	Emergency hospital admissions
Holland 2007 [25]	UK	RCT (1 INT, 1 C) *n* = 339	Post-discharge (after HF admission) Age: INT 77.6 (9); C 76.4 (9) Meds: INT 8 (3); C 8 (2)	2 visits (within 2 weeks, 6–8 weeks)	Educate, remove out of drugs and lifestyle behaviours, and provided BHF leaflet. Liaised with GP and local CP where needed	Usual care	Emergency hospital admissions
Jackson 2004 [28]	Australia	RCT (1 INT, 1 C) *n* = 128	Post-discharge and initiated on warfarin Age:*INT 70(−); *C 72.5(−) Meds: INT 6(−); C 6(−)	4 visits (2, 4, 6 and 8 days)	Education and counselling related to warfarin with booklet and info sheet provided. Post visit communication with GP	Usual care	Reduction in bleeding complications
Lenaghan 2007 [33]	UK	RCT (1 INT, 1 C) *n* = 136 *The POLYMED trial*	Community Age: INT 84.1(−); C 84.5 (−) Meds: INT 7(−); C 7(−)	2 visits (within 2 weeks, 6–8 weeks)	Medication review, educated the patient, removed out-of-date drugs, and assessed the need for an adherence aid. Post visit communications with GP	Usual care	Non elective hospital admissions
Naunton 2003 [32]	Australia	RCT: 1 INT, 1 C *n* = 136	Post-discharge with ≥2 chronic diseases Age: INT 74(−); C 74 (−) Meds: INT > 5(−); C > 5(−)	2 visits (5 days, 3 months)	Medication review, optimise medication management, education and detect DRPs	Usual care	Unplanned hospital admissions at 90 days, & Death
Olesen 2014 [26]	Denmark	RCT: 1 INT, 1 C *n* = 630	Community Age: *INT 74(−);*C 74(−); Meds: INT 7(−); C 7(−)	1 visit followed up by telephone calls at 3, 6 and 9 months	Medication review, counselling on medication, motivated adherence, provided leaflets. Contact with GP if serious issue.	Usual care	Medication adherence (Pill count)
Peterson 2004 [29]	Australia	RCT (1 INT, 1 C) *n* = 94	Post-discharge (after CV-related admission) and initiated on statins Age: INT 65.5 (11); C 63.5 (12) Meds:*8(−); *8(−)	1 visit at 6 weeks, and monthly thereafter up to 6 months	Educated on the goals and proven benefits of lipid-lowering drug therapy, and appropriate lifestyle modifications. Assessed for DRPs and compliance checked	Usual care	Cholesterol levels
Sidel 1990 [22]	USA	RCT (1 INT, 1 C) *n* = 284	Community at high risk of medication issues Age: INT > 65(−); C > 65(−) Meds: INT NR; C NR	2 visits over a 6–11 months with telephone follow-up as needed	Education on medication, remove out of date drugs, encourage communication with health providers if any issues arose.	Usual care	Medication management
Triller 2007 [30]	USA	RCT (1 INT, 1 C) *n* = 144	Post-discharge (after HF admission) Age: INT 81.3 (9); C 78.1 (11) Meds: INT NR; C NR	3 visits over 3–4 weeks	Medication review, counselling on medication, healthy lifestyle advice.	Usual care	All cause hospital admissions, HF- related admissions
Tuttle 2018 [31]	USA	RCT (1 INT, 1C) *n* = 159	Post-discharge (after CKD admission) Age: INT 70 (12); C 69 (10) Meds: ALL 13 (5)	1 visit within 7 days	Comprehensive medication review, medication action plan, and a personal medication list. Advice on proper medicine use and avoidance of contraindicated drugs. Post visit contact with GP if needed	Usual care	Acute care utilisation (hospital admissions and ED/ urgent care centre visits
Vuong 2008 [27]	Australia	RCT (1 INT, 1 C) *n* = 316	Post-discharge Age: INT 74.4 (11); C 69.3 (12); Meds: INT 11 (4); C 10 (4)	1 visit within 5 days	Medication reviews, education and information on medications, removal of our-of-date drugs. Post visit contact with GP and CP if needed.	Usual care	Medication adherence Medication knowledge

Key:

*RCT* Randomised controlled trial, *INT* Intervention, *C* Control, GP General Practitioner, *CP* Pharmacist, *HF* Heart failure, *CV* Cardiovascular, *DRP* Drug related problems, * medians; *NR* Not reported

### Study characteristics

The trials were conducted in the UK (*n* = 4), Australia (n = 4), the United States of America (*n* = 3), and Denmark (*n* = 1). The trials were primarily conducted between 2003 and 2015, with only two before that in 1990 [32] and 1995 [35]. All except one trial were RCTs with one intervention arm. Begley et al. [35] involved two intervention arms; home visits or home visits with medication education from the pharmacists. Trial size ranged from 94 to 822 participants, with five [22, 24, 26, 30, 32] of the 11 trials having > 250 participants. In total, 3410 participants were enrolled across the studies.

All of the trials involved populations considered at risk of medication-related problems: five involved populations with specific clinical health issues (heart failure, or new prescription of warfarin or statins, chronic kidney disease) recently discharged from hospital [26, 27, 31, 33, 34]; four involved elderly or older adult populations being discharged from hospital after emergency admission (not defined further) [23, 24, 29, 35]; and three involved elderly populations on multiple medications living in the community [28, 30, 32]. None of the trials were aimed solely at housebound individuals, but two specifically reported them as being included as part of the eligible population [23, 28]. Polypharmacy was a requirement of entry in seven of the trials: four included individuals taking a minimum of two or three medications [23, 24, 26, 35], and three including individuals taking four medications or more [28–30]. At baseline, eight studies reported participants taking a mean of 5 to 9 medicines per day, and two studies, over > 10 medications [23, 34]. In ten of the 12 trials, the mean/median age of the recruited population was 70 years or more.

### Intervention characteristics

The purpose, frequency, and content of the intervention varied considerably. Eight studies specifically reported carrying out a ‘*medication review’* as a purpose of the home visit, and went on to describe components of the review such as removal of out of date drugs, as well as providing education and information on medications [23, 24, 26, 28–30, 34]. For the four trials that did not specifically use the term ‘*medication review’*, three described processes akin to a review, with the purpose of the home visit being to educate, remove out of date drugs, assess adherence and advise on medication issues [31, 32, 35], and the remaining study was specific to warfarin management [27]. The principal aim of the home visit intervention as reported was to reduce hospital admission in six studies [24, 26, 28, 29, 33, 34]) to improve adherence and medication management in four studies [23, 30, 32, 35], and to reduce medication issues in two studies [27, 31]. The number of home visits made by pharmacists ranged from one to six visits, over periods ranging from 2 weeks to 1 year. The most intense pharmacist intervention, in terms of house visits, was four visits over the course of 8 days [27], and the least intense studies included two visits over the course of 11 months [32] and single visits [23, 30, 34]. Follow-up with the participant over the telephone was reported to be part of the routine intervention for only two studies [30, 32]. Five studies [23, 27–29, 31] reported routinely liaising with relevant health professionals (GP, local pharmacist) after the home visit, four studies [24, 26, 30, 34] reported contacting health professionals as required, and three made no mention of post visit contacts [32, 33, 35]. For all studies, the control group was usual care.

The number of pharmacists per trial and pharmacist experience, training and employment also varied. Three studies used a team of pharmacists, with two providing 1 to 2 day training [24, 26] on the purpose and nature of the intervention, and one providing no training [30]. One study employed two full time clinical pharmacists specifically for the trial [23], and reported using detailed protocols were used for the home visits. Six studies [27–29, 31, 33, 35] reported using a sole project/research pharmacist for the duration of the trial, only one of which reported on the suitability of experience of the pharmacist for the home visit role [33]. Two studies provided no information on the number or experience of pharmacists conducting the intervention [32, 34].

### Outcomes

Six of the studies reported rate of hospitalisation as the primary outcome [24, 26–29, 33] and two as a secondary outcome [30, 34]. Mortality was reported as a primary outcome in one study [29] and as a secondary outcome in seven [26–30, 33, 34]. For both these outcomes (rate of hospital admission and mortality), there were differences in follow-up time, with three studies reporting outcomes at 3 months, four studies at 6 months, and one after 2 years. Care home admission was reported in two studies, both at 6 months follow up. Medication adherence was reported as a primary outcome in two studies [25, 35], and as a secondary outcome in five studies [26, 29, 31]. Adherence was measured in a variety of ways: through a variety of subjective self–report measures and in a couple of studies pill count [29, 30]. Quality of life was assessed as a secondary outcome in four studies [24, 26, 28, 33]. Three studies used the EQ. 5D tool, and one study used a bespoke survey [33]. The EQ. 5D includes a visual analog health scale (VAHS) where people rate their overall health in the last month from 100 (perfect health) to 0 (worst imaginable health). Medication knowledge, measured in two studies [23, 35], and medication hoarding, measured in one study [35], were measured using a bespoke questionnaire.

### Risk of bias

**Fig. 2:**
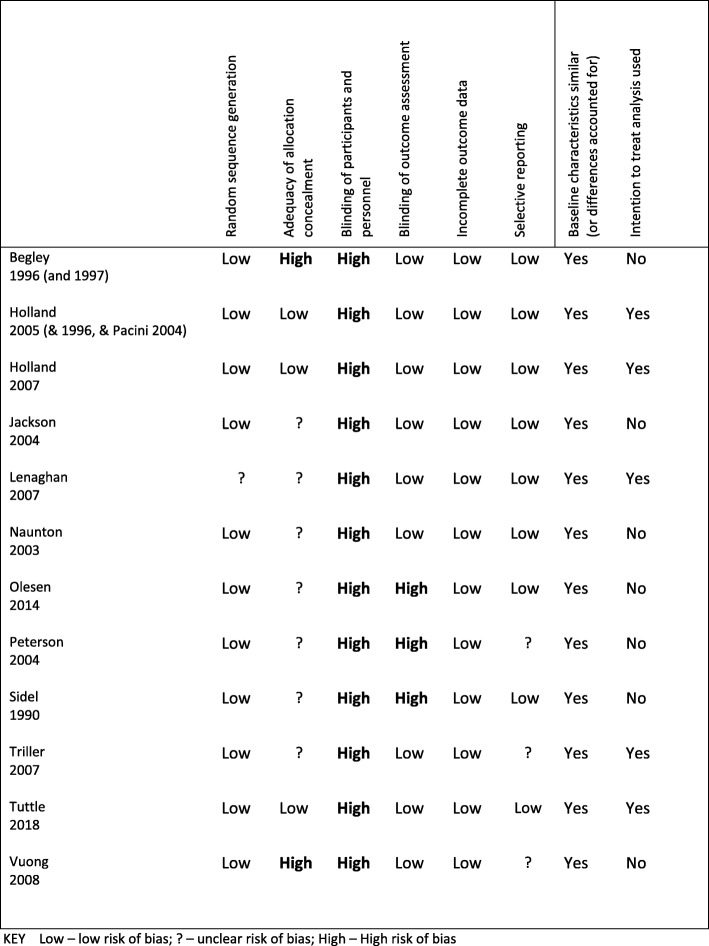
Cochrane risk of bias summary for included studies

A summary of the risk of bias is presented in Fig. 2. Despite all being described as RCTs, the method of random sequence generation was not reported in three studies. Most studies did not report sufficient detail to assess whether allocation concealment was adequate. Due to the nature of the intervention, all of the studies were at high risk of performance bias with participants and pharmacist clearly aware of the group allocation. Detection bias was considered low for studies reporting the primary outcome measure of hospital admission or mortality statistics, but considered high for the studies reporting the primary outcome measure as medication adherence with measurement from self-report. Across most of the studies, reporting bias was low with outcome data complete or missing data accounted for adequately. Only three studies [24, 26] were rated as being at a low risk of bias across all domains, excluding blinding of participants and study personnel.

The principal findings from the 12 studies are detailed in Table 2.

**Table 2:** Summary of study outcomes

Study	Number	Reported outcome measures	Results: Pharmacist home visit group compared to control
Begley 1995 [35] & Begley 1997 [23]	*n* = 222	Medication adherence at 12/12 Contact with GPs from 3/12 to 12/12 Hoarding at 3/12 Economic evaluation	Improved: 86% vs 69%, *p* = 0.0001 Less: 54% vs 74%, *p* < 0.01 Less: 1% vs > 95%, *P* < 0.001 Cost effective (net benefit of the 1st visit £864.47 dropping to £4.87 at the 5th visit)
Holland 2005 [24] & Pacini 2007 [5]	*n* = 822	Emergency hospital readmission over 6/12 Death within 6/12 Admission to care home over 6/12 QOL EQ. 5D change over 6/12 QOL VAHS change over 6/12 Hoarding Economic evaluation	RR 1.30, 95%CI (1.07 to 1.58), *p* = 0.0009 HR 0.75 (0.53 to 1.10), *p* = 0.14 37/300 vs 32/285, NS 0.006 (− 0.048 to 0.059), *p* = 0.84 Worse: −4.12 (−8.09 to − 0.15), *p* = 0.042 Decreased in INT: 40 to 19%, *p* < 0.001 Not cost effective (net increase of £271 for NHS per patient)
Holland 2007 [25]	*n* = 339	Emergency hospital readmission over 6/12 Death within 6/12 QOL EQ. 5D change over 6/12 QOL VAHS change over 6/12 Medication adherence (MARS) at 6/12	RR 1.15 (1.08 to 1.40), *p* = 0.59 HR 1.18 (0.69 to 2.03), *p* = 0.54 0.07 (− 0.01 to 0.14), *p* = 0.08 − 0.93 (− 6.05 to 4.20), *p* = 0.72 0.12 (− 48 to 0.73)*p* = 0.68
Jackson 2004 [28]	*n* = 128	Bleeding complications over 3/12 Readmission due to bleeding over 3/12 Death within 3/12	15% vs 36%, *p* = 0.009 3% vs 8%, *p* = 0.32 7% vs 8%, *p* = 0.90
Lenaghan 2007 [33]	*n* = 136	Non elective hospital admissions over 6/12 Death within 6/12 (% diff in proportion) Admission to care home over 6/12 (% diff in proportion) QOL EQ. 5D change over 6/12 QOL VAHS change over 6/12	RR 0.92 (0.50 to 1.70), *p* = 0.80 1.3% (− 12.2 to 14.7), *p* = 0.81 − 3.0% (− 11.0 to 5.0), *p* = 0.30 0.09 (− 0.19 to 0.02), *p* = 0.10 4.8 (− 12.5 to 2.8), *p* = 0.21
Naunton 2003 [32]	*n* = 136	Unplanned hospital admissions at 3/12 Death within 3/12 Medication adherence (“never miss”)	28% vs 45%, *p* = 0.05 5% vs 8%, not reported 87% vs 44%, *p* < 0.001
Olesen 2014 [26]	*n* = 630	Non elective hospital admissions over 24/12 Death within 24/12 Medication adherence (% non-adherent)	OR 1.14 (0.78 to 1.67), NS HR 1.41 (0.71 to 2.82), NS 11% vs 10%, NS
Peterson 2004 [29]	*n* = 94	Medication adherence (never/rarely miss) Cholesterol levels	NS difference 4.4 (0.6) vs 4.6 (0.8) mmol/L, *p* = 0.24
Sidel 1990 [22]	*n* = 284	Medication adherence (change in those who ‘remember to take’) Health service contact (change in 3/12)	−.09 vs − 0.19, *p* = 0.52 − 1.16 vs 0.25, *p* = 0.08
Triller 2007 [30]	*n* = 144	All cause hospital admissions, HF- related admissions Death QOL (not named) Care costs	58% vs 55%, *p* = 0.63 51% vs 42%, *p* = 0.26 18% vs 22%, *p* = 0.67 NS difference NS difference
Tuttle 2018 [31]	*n* = 159	Acute care utilisation (hospital admissions, emergency care visits, urgent care centre visits)	NS difference: 44% vs 41%, *p* = 0.72 (hospital admissions 26% vs 26%, *p* = 0.95)
Vuong 2008 [27]	*n* = 316	Medication adherence (modified Morisky) Medication knowledge (bespoke)	Improved: 0.23 vs 0.41, *p* = 0.028 Improved: 0.70 (0.24) vs 0.78 (0.14), *p* = 0.001

Key: *QOL* Quality of Life, *RR* Rate ratio, *OR* Odds ratio, *HR* Hazard ratio, *NS* Not significant, *HF* Heart failure

### Effects of intervention on hospital admissions and mortality

**Fig. 3:**
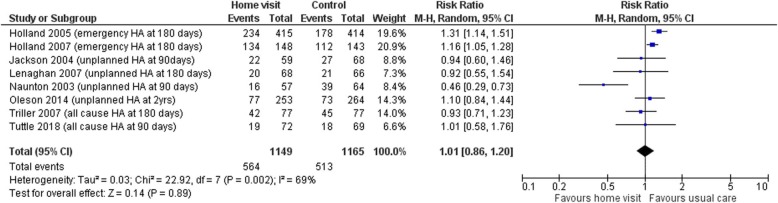
Forest plot of the pooled analyses showing the risk ratio for hospital admissions with pharmacist home visit intervention compared to usual care

**Fig. 4:**
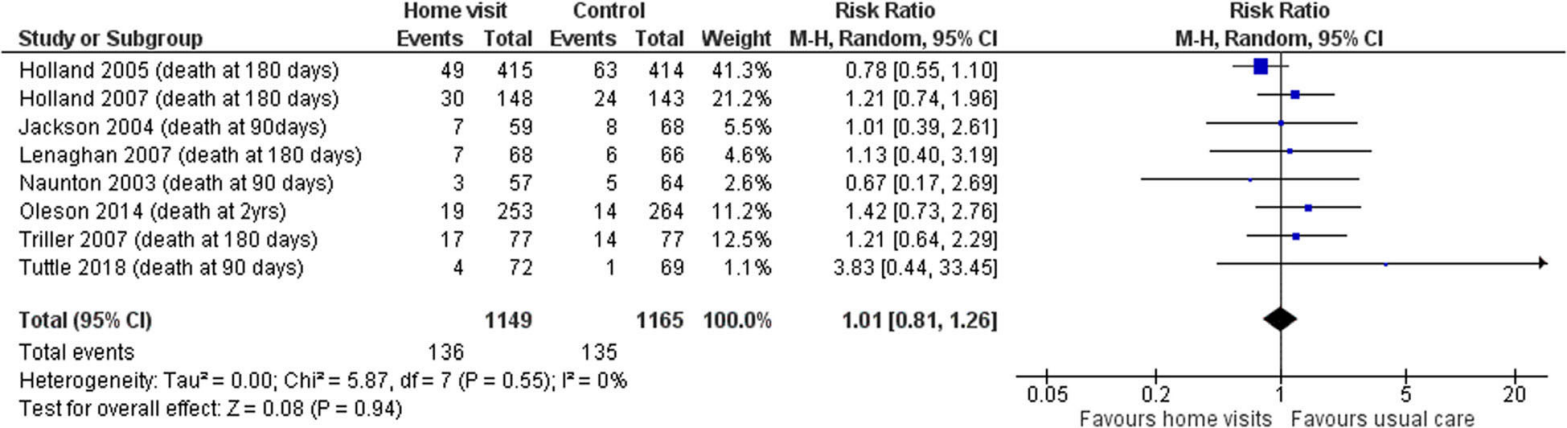
Forest plot of the pooled analyses showing the risk ratio mortality with pharmacist home visit intervention compared to usual care

Data from all eight trials measuring hospital admissions and mortality data were included in meta-analyses. Hospital admissions were either described as unplanned admissions, emergency admissions or total admissions. There was no evidence of a significant effect of intervention for either outcome. The pooled relative risk (RR) of hospital admission for those receiving home visits compared to those under usual care was 1.01 (95%CI 0.86 to 1.20, I^2^ = 69.0%, *p* = 0.89; 8 studies, 2314 participants). The high level of heterogeneity found here is mostly explained by the study of Naunton et al. [29] which was the only study to report a significant reduction in admissions in the group who received home visits compared to usual care (28% compared to 45%, *p* < 0.05). The pooled RR for death was 1.01 (95%CI 0.81 to 1.26, I^2^ = 0%, *p* = 0.94; 8 studies, 2314 participants). The forest plots for these analyses are shown in Figs. 3 and 4.

### Effects on care home admission

Two studies reported on care home admissions as secondary outcomes [24, 28]. Neither study found any effect of pharmacist home visit intervention compared to usual care. In the HOMER trial [24], 21 out of 429 (7%) home visit participants were admitted to care homes over the 6 months compared to 17 out of 426 (6%) in the control group. The difference in proportions was not significant (95% CI − 3.1 to 5.2%, *p* = 0.61). In the trial by Lenaghan et al. [28], one person of 69 (1.5%) in the home visit group entered a care home over the 6 months compared to 3 of 67 (4.5%) in the control group. Again the difference in proportions was not significant (95%CI − 11.0 to 5.0%, *p* = 0.30).

### Effects of intervention on quality of life

**Fig. 5:**
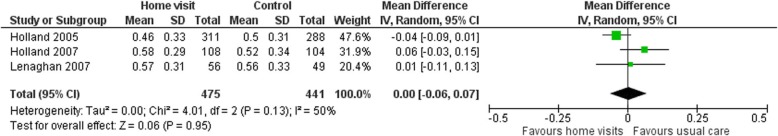
Forest plot of the pooled analyses showing the effect of pharmacist home visit compared to usual care on quality of life measured by utilities scores of the EQ. 5D

**Fig. 6:**
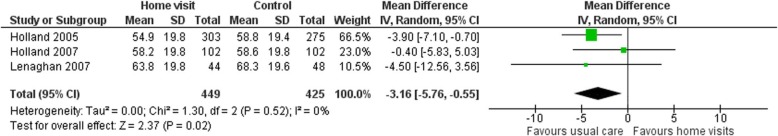
Forest plot of the pooled analyses showing the effect of pharmacist home visit compared to usual care on quality of life measured by the VAHS (EQ. 5D)

Four studies measured quality of life as an outcome. Data from three studies using the EQ-5D to assess quality of life could be pooled [24, 26, 28]. There was no evidence of effect on quality of life as measured by the utility scores of the EQ-5D (see Fig. 5). The pooled standardised mean difference (SMD) was 0.01 (95%CI − 0.20 to 0.22, I^2^ = 0%, *p* = 0.94; 3 studies, 916 participants)). There was, however, evidence suggesting a small negative effect of home visits compared to usual care for participants self-rated health as measured by the VAHS component of the EQ-5D (SMD of − 0.16 (95%CI − 0.29 to − 0.02, I^2^ = 0%, *p* = 0.02; 3 studies, 916 participants), see Fig. 6. The study by Triller et al. [33] did not present any raw data, but reported no difference in quality of life as measured by a survey (specific tool not reported) between those who had received visits compared to those who had not.

### Effects of intervention on medication adherence

Seven studies assessed medication adherence ([23, 26, 29–32, 35]).However, due to the variety of adherence assessment and insufficient details (no SDs, no raw data), we were unable to pool data. The findings across the studies were inconsistent, with three studies (two self-report [23, 35] and one using objective assessment [29]) reporting significant improvement in adherence after home visit intervention and four studies (one objective [30] and 3 using self-report measures [26, 31, 32]) finding no effect of intervention. Begley and colleagues [35] reported 86% of the intervention group fully compliant at 1 year compared to 69% of group who had received no home visits (*p* < 0.001). Voung and colleagues [23] reported improvements in adherence, measured on the modified Morisky Scale in which the lower the number the better, in both arms after 12 weeks, but significantly greater improvement in those who received home visits (0.4 to 0.23 for the intervention, compared to 0.55 to 0.41 for the control, *p* < 0.028). Pill count observations in the study by Naunton et al. showed 5% of the intervention group to be non-compliant compared to 22% of the control group (*p* < 0.01) after 3 months, and this was further supported by significantly improved self-report compliance [29].

In contrast, Peterson et al. [31] in their study of patients recently discharged from hospital after a cardiovascular related event found no difference in those who said they either never or rarely missed their medication between the trial arms (raw data not shown) at trial end at 6 months. Likewise, Sidel et al. [32] reported no significant difference between home visits and usual care in those reporting that they remembered to take meds, or those stopping meds without telling their physician. Holland et al. [26] found no difference in MARS scores between intervention and control in the recently discharged population of the HOMER study. Oleson and colleagues ([30]) in their study of the elderly living in the community found nonadherence rates, measured using a pill counter pen, of 11% in those receiving visits compared to 10% of those in usual care at the trial end at 1 year.

### Effects of intervention on knowledge and hoarding

Two studies measured the effect of pharmacist home visits on medication knowledge [23, 35]. Begley ([35] asked patients about the name, purpose, dosage, frequency and duration of each of their prescribed and purchased drugs and were scored on their percentage correctness. Whilst they observed a significant improvement in knowledge in the intervention group at 2 weeks compared to their baseline scores (after one visit), they found no significant difference at 12 months between those who had received visits (five visits over the year) compared to those who had received no visits (70% compared to 66%, respectively - no stats provided). Vuong and colleagues also used a composite measure of knowledge about name, dose, frequency, strength, indications and side effects, and their total score was divided by the total possible score, giving a range of 0.0–1.0 possible, and a score of 0.75 or more deemed good medication knowledge [23]. At 8 weeks, medication knowledge was higher in the control group compared to those who had received a pharmacist visit in the first 2 weeks (0.78 compared to 0.70, *p* < 0.001). Baseline scores were not reported so it is not known whether there was a difference to begin with.

The only two studies to report on hoarding (drugs found that were either out of date, duplicated or no longer necessary) both found significant reductions. Hoarding decreased from 61% at baseline to 5% at 12 months in those receiving home visits, compared to little change from 98 to 95% in control patients (*p* < 0.001) in the study by Begley [35], and was reduced in the home visit group from 40 to 19% at 2 months (control group data not reported) in the study by Holland and colleagues [34].

### Effects of intervention on healthcare costs

Three studies reported the estimated cost of a pharmacist home visit intervention with mixed findings. Pooling of the data was not possible. Begley [35] found the increase in benefits outweighed any increase in costs. The estimated net cost savings were between £216 and £26,840 for the 61 intervention participants. Marginal analysis demonstrated that the net benefit of providing a fifth visit to each patient was only £4.87 compared to the net benefit of the first visit (£864.47). In contrast, a detailed economic evaluation by Pacini et al. [5] found a net increase in cost to the NHS of £271 per patient in the HOMER trial. Triller et al. [30] reported no difference in total aggregate healthcare costs, hospital costs or home care agency costs between those in the home visit group compared to those receiving usual care.

## Discussion

This is the first systematic review to evaluate the effectiveness of home visits by pharmacists for people at risk of medication-related problems. Twelve RCTs were included, and the effects on hospital admissions, mortality, quality of life, medication adherence, medication knowledge and costs were assessed. The review found no evidence of effect on hospital admission or mortality rates. There was also no effect on care home admissions in the two studies that reported on this. The review findings also suggest no consistent benefit on medication adherence and knowledge or quality of life, and limited evidence of cost effectiveness. None of the included studies explored whether there were different effects for the older or more vulnerable subgroups of the population studied.

Despite differences in target populations, and frequency and purpose of intervention, the findings of no beneficial effect were fairly consistent across the included studies. The exception to this was the hospital admission data, in which the considerable heterogeneity is mostly explained by the study of Naunton and colleagues [32]. In this relatively small study of 122 discharged elderly patients, hospital admissions were significantly lower in the home visit group, and this was the only study of the eight to find this, with two larger studies finding the opposite [24, 25]. In this trial, the baseline characteristics of the population was comparable to the other studies: older adults with a mean age of 75 yrs. taking on average 8 prescription drugs, and 40–50% living alone. The intervention consisted solely of one visit at 5 days post discharge, but a full medication review was undertaken at this visit, a summary of recommendations left with the participant and the findings were routinely communicated by phone to both the general practitioner and the local pharmacist. The lack of ‘inter-professional communication’ has been postulated as one of the key factors for why pharmacist-led home visits to date have not been found to be more successful [26, 34, 36]. The study by Naunton and colleagues was one of only two studies which reported that the pharmacist spoke to the GP after every participant visit. The only other study reporting spoken contact [33], involved regular face to face meetings between pharmacist and GP, but found no effect on hospital admissions. Another factor which may help explain the benefit found in the study by Naunton and colleagues was that the intervention was facilitated by a single study-specific pharmacist recruiting from one hospital. Whilst this does impact on its generalisability, it did perhaps allow for a well-resourced focus on the intervention. In many of the other included studies in this review, the pharmacist(s) was performing the home visit in addition to their normal work routine. The final factor which needs to be considered is the short follow-up. Naunton et al. was one of only three studies assessing hospital admissions at 3 months post intervention, with the remaining five studies having follow-up of 6 months to 2 years. Shorter as opposed to longer follow up was also associated with greater reductions in all cause hospital admissions in a recent review of pharmacist reconciliation programmes at hospital transition [37].

It is also perhaps unsurprising that no evidence of benefit on hospital admissions or mortality was observed as there was little evidence of a consistent benefit on medication adherence or knowledge. Four of seven studies that measured it found no improvement in adherence, though the measures used to assess adherence were largely not objective. Of interest, of the three studies that reported greater medication adherence, two reported significant improvements in clinical outcome, Begley [23] finding less contact with GPs over 12 months in those who had received up to five pharmacist home visits, and Naunton [32] (as discussed above), fewer unplanned hospital admissions. The third study to find greater adherence did not assess any measure of healthcare contact. These studies are in accordance with a recent Cochrane review which concluded that current methods of improving adherence in chronic health problems are mostly complex and not very effective [38].

There was no evidence of improvement in quality of life with pharmacist home visit intervention, and a suggestion that the participants in the home visit group had slightly lower perceived health as measured by the VAHS part of the EQ. 5D, despite no difference in the index score. Whilst data for the meta-analysis were obtained from only three studies, the studies were of reasonable size and trials assessed as at a low risk of bias. Without a qualitative exploration of what participants felt about the pharmacist home intervention it is difficult to interpret this possible negative effect. A few studies reported that the intervention was well received [23, 25, 29, 32], but it may be that participants felt more unsettled either having another professional visit them in their home, or not their regular pharmacist, or the visit highlighted their vulnerability in some way. Indeed Holland et al. [24] in trying to explain their findings of increased hospital admissions in their intervention group, suggested that home visits might have led participants to be more aware and ruminate about possible warning signs, which could make people more anxious about their health. The finding that there was a change in the VAHS score but not index score was a little surprising, but a lack of correspondence between the VAHS and the EQ. 5D utility score has been observed before [39]. Possible explanations include the difference in reporting numerically versus spatially, that the VAHS response is influenced not only by participant state of health but also by personal characteristics, such as psychological disposition, age, sex, education; and that the utility score is informed by opinions/comparisons with how members of the public perceive health disutility, as opposed to the VAHS which is completed by people experiencing health [39].

## How does this compare review compare to others

These results compare well with previous systematic reviews in related areas [12, 13, 38, 40]. The meta-analysis of pharmacist-led medication reviews of people > 60 years in any setting [13] found no significant effect on either all-cause admission or mortality, with RR values comparable with those found in our review. Whilst larger in scope, our findings also match those of the recent systematic review of preventative home visiting by a health or social care professional of community dwelling older adults [12]. This meta-analysis, which included 64 RCTs, concluded that although preventative home visiting was associated with a small decrease in relative risk of mortality (RR = 0.93 [95% CI 0.87–0.99], the absolute reduction was close to zero. Furthermore, they found no evidence of benefit from preventative home visits for institutionalisation or hospitalisation and only low quality evidence for benefits on quality of life. However, as there was scant detail on how well each intervention was delivered, the authors postulated that maybe the lack of evidence of effect might have been due to interventions not being delivered as intended, rather than the interventions per se being ineffective [12].

## Strengths and limitations

This systematic review followed best practice. We searched all key, relevant databases and supplemented the database searches with forward and backward citation searching. We acknowledge that there may be unpublished data that we have not been able to retrieve, but given the number of studies already in our meta-analysis, these results are likely to be significantly changed only if the unpublished studies consisted of several large scale RCTs. We attempted to evaluate patient related measures such as medication-related problems, adherence and quality of life, in addition to outcomes such as hospital admissions and mortality which may not relate specifically to the interventions delivered, or a particular patient’s needs. However, these other outcomes were not consistently reported, thus limiting our ability to draw any robust conclusions on the effectiveness of the intervention on such outcomes.

## Implications for research and practice

Many of the included studies in this review chose hospital admissions and mortality as the primary outcome, but whether they are suitably sensitive for the interventions undertaken has been questioned and has their relevance [41, 42]. Indeed medication related hospital admission might be more appropriate. Further, despite many authors highlighting the wide-scale problem of drug related issues as part of the rationale for their study, only two studies assessed the effect of the intervention on them. Having more robust measures of medication adherence, medication knowledge and changes in drug-related problems may be worth exploring. Some have suggested that use of a core outcome set (COS) for studies of medication review in older people would reduce the heterogeneity between trials, increase research output that has relevant outcomes and result in more usable data to inform evidence synthesis [42]. Whilst a couple of COSs have recently been developed [43, 44], they will only have impact if used consistently in research trials.

Qualitative exploration of what participants at risk of medication issues feel they need to improve their adherence and understanding is important along with their preferences regarding home visits per se. How to improve collaboration and establishment of trust between those at home, the pharmacist and general practitioner has also been recognised as an important area to investigate [45]. Lastly, whilst the populations in the included studies were predominantly > 70 yrs., and most were taking five medications or more, there remains considerable heterogeneity amongst such populations. There may be merit in focussing on home visits for the purposes of medication management in the more vulnerable: those > 80 years, living alone, isolated or housebound or at the high end of polypharmacy, especially since the number of adults > 65 years prescribed 10 or more medications has tripled in recent years [46]. For these populations, interventions that focus on helping the carer and/or paid carers in their understanding and appropriate use of medications need researching [47]. This may be particularly important for maintaining older adults in their home, an outcome which was only reported on by two studies in this review. Further research in this area is much needed.

## Conclusion

This systematic review found no evidence that pharmacist-led medicine reviews in the home for individuals at risk of medication-related problems reduced hospital admissions or mortality. If the aim of studies investigating medicine reviews is to improve medication literacy and adherence, with the effect of reducing the number of drug related problems, then greater investment in robust methods to assess relevant outcomes is needed. Future studies undertaken in this area may also benefit from targeting specifically vulnerable populations and use approaches that maximise collaboration and communication between the participant, pharmacist, and the primary care physician.

## Supplementary information

**Additional file 1.** Example search strategy for OVID Medline. Search strategy.

## Data Availability

Not applicable, as this research article reviewed the published literature. The data published may be found in the original manuscripts cited in the references list.

## Abbreviations

CI: Confidence intervals
COS: Core outcome set
CP: Community pharmacist
DRP: Drug related problems
EQ-5D: EuroQol- 5 dimension
GP: General practitioner
HR: Hazard ratio
MARS: Medication adherence rating scale
NIHR: National institute for health and care excellence
OR: Odds ratio
PRISMA: Preferred reporting items for systematic reviews and meta analyses
QOL: Quality of life
RCT: Randomised controlled trial
RR: Relative risk
SD: Standard deviation
SMD: Standardised mean difference
UK: United Kingdom
VAHS: Visual analogue health scale (EQ-5D)

## References

[1] O'Quin KE, Semalulu T, Orom H (2015). Elder and caregiver solutions to improve medication adherence. Health Educ Res.

[2] Somers A, Robays H, Vander Stichele R, Van Maele G, Bogaert M, Petrovic M (2010). Contribution of drug related problems to hospital admission in the elderly. J Nutr Health Aging.

[3] Cahir C, Curran C, Byrne C, Walsh C, Hickey A, Williams DJ, Bennett K (2017). Adverse Drug reactions in an Ageing PopulaTion (ADAPT) study protocol: a cross-sectional and prospective cohort study of hospital admissions related to adverse drug reactions in older patients. BMJ Open.

[4] Department of Health (2001). National Service Framework for Older People.

[5] Pacini M, Smith RD, Wilson EC, Holland R (2007). Home-based medication review in older people: is it cost effective?. Pharmacoeconomics.

[6] Health Do (2005). The Pharmaceutical Services (Advanced and Enhanced Services) (England) Directions. In.

[7] Latif A, Pollock K, Boardman HF (2013). Medicines use reviews: a potential resource or lost opportunity for general practice?. BMC Fam Pract.

[8] Chen TF (2016). Pharmacist-Led Home Medicines Review and Residential Medication Management Review: The Australian Model. Drugs Aging.

[9] Oxley D (1999). Providing a pharmacy service to the over 75s – need they be visited at home?. Pharm J.

[10] Cohen-Mansfield J, Shmotkin D, Hazan H (2012). Homebound older persons: prevalence, characteristics, and longitudinal predictors. Arch Gerontol Geriatr.

[11] Herr M, Latouche A, Ankri J (2013). Homebound status increases death risk within two years in the elderly: results from a national longitudinal survey. Arch Gerontol Geriatr.

[12] Mayo-Wilson E, Grant S, Burton J, Parsons A, Underhill K, Montgomery P (2014). Preventive home visits for mortality, morbidity, and institutionalization in older adults: a systematic review and meta-analysis. PLoS One.

[13] Holland R, Desborough J, Goodyer L, Hall S, Wright D, Loke YK (2008). Does pharmacist-led medication review help to reduce hospital admissions and deaths in older people? A systematic review and meta-analysis. Br J Clin Pharmacol.

[14] Nazar H, Nazar Z, Portlock J, Todd A, Slight SP (2015). A systematic review of the role of community pharmacies in improving the transition from secondary to primary care. Br J Clin Pharmacol.

[15] Renaudin P, Boyer L, Esteve MA, Bertault-Peres P, Auquier P, Honore S (2016). Do pharmacist-led medication reviews in hospitals help reduce hospital readmissions? A systematic review and meta-analysis. Br J Clin Pharmacol.

[16] Gowing A, Robinson L (2015). Home visits and the housebound. InnovAiT Educ Inspriation Gen Pract.

[17] Centre for Reviews and Dissemination (2009). Systematic Reviews: CRD's Guidance for Undertaking Reviews in Healthcare.

[18] Moher D, Liberati A, Tetzlaff J, Altman DG, Group P (2009). Preferred reporting items for systematic reviews and meta-analyses: the PRISMA statement. J Clin Epidemiol.

[19] Higgins JP, Altman DG, Gotzsche PC, Juni P, Moher D, Oxman AD, Savovic J, Schulz KF, Weeks L, Sterne JA (2011). The Cochrane Collaboration's tool for assessing risk of bias in randomised trials. BMJ.

[20] Hedges LV (1985). OI: Statistical methods for meta-analysis.

[21] Higgins JP, Thompson SG, Deeks JJ, Altman DG (2003). Measuring inconsistency in meta-analyses. BMJ.

[22] Sidel VW, Beizer JL, Lisi-Fazio D, Kleinmann K, Wenston J, Thomas C, Kelman HR (1990). Controlled study of the impact of educational home visits by pharmacists to high-risk older patients. J Community Health.

[23] Begley S, Livingstone C, Hodges N, Williamson V (1997). Impact of domiciliary pharmacy visits on medication management in an elderly population. Int J Pharm Pract.

[24] Holland R, Lenaghan E, Harvey I, Smith R, Shepstone L, Lipp A, Christou M, Evans D, Hand C (2005). Does home based medication review keep older people out of hospital? The HOMER randomised controlled trial. BMJ.

[25] Holland R, Brooksby I, Lenaghan E, Ashton K, Hay L, Smith R, Shepstone L, Lipp A, Daly C, Howe A (2007). Effectiveness of visits from community pharmacists for patients with heart failure: HeartMed randomised controlled trial. BMJ.

[26] Olesen C, Harbig P, Buus KM, Barat I, Damsgaard EM (2014). Impact of pharmaceutical care on adherence, hospitalisations and mortality in elderly patients. Int J Clin Pharm.

[27] Vuong T, Marriott JL, Kong DCM, Siderov J (2008). Implementation of a community liaison pharmacy service: A randomised controlled trial. Int J Pharm Pract.

[28] Jackson SL, Peterson GM, Vial JH, Jupe DM (2004). Improving the outcomes of anticoagulation: an evaluation of home follow-up of warfarin initiation. J Intern Med.

[29] Peterson GM, Fitzmaurice KD, Naunton M, Vial JH, Stewart K, Krum H (2004). Impact of pharmacist-conducted home visits on the outcomes of lipid-lowering drug therapy. J Clin Pharm Ther.

[30] Triller DM, Hamilton RA (2007). Effect of pharmaceutical care services on outcomes for home care patients with heart failure. Am J Health-Syst Pharm.

[31] Tuttle KR, Alicic RZ, Short RA, Neumiller JJ, Gates BJ, Daratha KB, Barbosa-Leiker C, McPherson SM, Chaytor NS, Dieter BP (2018). Medication therapy management after hospitalization in CKD: a randomized clinical trial. Clin J Am Soc Nephrol.

[32] Naunton M, Peterson GM (2003). Evaluation of Home-Based Follow-Up of High-Risk Elderly Patients Discharged from Hospital. J Pharm Pract Res.

[33] Lenaghan E, Holland R, Brooks A (2007). Home-based medication review in a high risk elderly population in primary care--the POLYMED randomised controlled trial. Age Ageing.

[34] Holland R, Lenaghan E, Smith R, Lipp A, Christou M, Evans D, Harvey I (2006). Delivering a home-based medication review, process measures from the HOMER randomised controlled trial. Int J Pharm Pract.

[35] Begley S. The establishment and evaluation of a domiciliary pharmacy service: University of Brighton; 1995.

[36] Kwint HF, Bermingham L, Faber A, Gussekloo J, Bouvy ML (2013). The relationship between the extent of collaboration of general practitioners and pharmacists and the implementation of recommendations arising from medication review: a systematic review. Drugs Aging.

[37] Mekonnen AB, McLachlan AJ, Brien JA (2016). Pharmacy-led medication reconciliation programmes at hospital transitions: a systematic review and meta-analysis. J Clin Pharm Ther.

[38] Nieuwlaat R, Wilczynski N, Navarro T, Hobson N, Jeffery R, Keepanasseril A, Agoritsas T, Mistry N, Iorio A, Jack S, et al. Interventions for enhancing medication adherence (Review). Cochrane Database Syst Rev. 2014;(11). 10.1002/14651858.CD000011.pub4 PMC7263418 25412402

[39] Whynes DK, McCahon RA, Ravenscroft A, Hodgkinson V, Evley R, Hardman JG (2013). Responsiveness of the EQ-5D health-related quality-of-life instrument in assessing low back pain. Value Health.

[40] Thomas R, Huntley AL, Mann M, Huws D, Elwyn G, Paranjothy S, Purdy S (2014). Pharmacist-led interventions to reduce unplanned admissions for older people: a systematic review and meta-analysis of randomised controlled trials. Age Ageing.

[41] Krska J, Hansford D, Seymour DG, Farquharson J (2007). Is hospital admission a sufficiently sensitive outcome measure for evaluating medication review services? A descriptive anaylysis of admissions within a randomised controlled trial. Int J Pharm Pract.

[42] Beuscart JB, Pont LG, Thevelin S, Boland B, Dalleur O, Rutjes AWS, Westbrook JI, Spinewine A (2017). A systematic review of the outcomes reported in trials of medication review in older patients: the need for a core outcome set. Br J Clin Pharmacol.

[43] Beuscart JB, Knol W, Cullinan S, Schneider C, Dalleur O, Boland B, Thevelin S, Jansen PAF, O'Mahony D, Rodondi N (2018). International core outcome set for clinical trials of medication review in multi-morbid older patients with polypharmacy. BMC Med.

[44] Rankin A, Cadogan CA, In Ryan C, Clyne B, Smith SM, Hughes CM (2018). Core Outcome Set for Trials Aimed at Improving the Appropriateness of Polypharmacy in Older People in Primary Care. J Am Geriatr Soc.

[45] Loffler C, Koudmani C, Bohmer F, Paschka SD, Hock J, Drewelow E, Stremme M, Stahlhacke B, Altiner A (2017). Perceptions of interprofessional collaboration of general practitioners and community pharmacists - a qualitative study. BMC Health Serv Res.

[46] Molokhia M, Majeed A (2017). Current and future perspectives on the management of polypharmacy. BMC Fam Pract.

[47] Wagle KC, Skopelja EN, Campbell NL (2018). Caregiver-Based Interventions to Optimize Medication Safety in Vulnerable Elderly Adults: A Systematic Evidence-Based Review. J Am Geriatr Soc.

