# Supplementary material for: Effectiveness of pharmacist home visits for individuals at risk of medication-related problems: a systematic review and meta-analysis of randomised controlled trials
Source: BMC Health Serv Res. 2020 Jan 15;20:39. doi: 10.1186/s12913-019-4728-3 (PMC6961241; doi:10.1186/s12913-019-4728-3)
Supplement: Supplementary file 1 — Additional file 1. Example search strategy for OVID Medline. Search strategy. [file 12913_2019_4728_MOESM1_ESM.doc]

Database: Ovid MEDLINE(R) In-Process & Other Non-Indexed Citations and Ovid MEDLINE(R) <1946 to Present>

Search Strategy:

--------------------------------------------------------------------------------

1 exp Community Pharmacy Services/ (3015)

2 Pharmacists/ (11288)

3 Pharmaceutical Services/ (4377)

4 (pharmacy or pharmacist*).ti,ab. (41641)

5 1 or 2 or 3 or 4 (46311)

6 Home Care Services/ (28227)

7 House Calls/ (2555)

8 (house* and (visit* or call* or appointment*)).ti,ab. (8114)

9 (home* and (visit* or call* or appointment*)).ti,ab. (23570)

10 domiciliary.ti,ab. (2256)

11 6 or 7 or 8 or 9 or 10 (58053)

12 5 and 11 (804)

13 medication* review*.ti,ab. (663)

14 drug* review*.ti,ab. (338)

15 medicine* review*.ti,ab. (385)

16 pharmaceutical review*.ti,ab. (22)

17 (review* adj2 pharmacist*).ti,ab. (342)

18 13 or 14 or 15 or 16 or 17 (1655)

19 (general adj2 practice*).ti,ab. (37059)

20 primary care.ti,ab. (76505)

21 GP* surger*.ti,ab. (288)

22 19 or 20 or 21 (108037)

23 5 and 18 and 22 (104)

24 12 or 23 (895)

***************************
